# Pain-related disability in functional neurological disorder (FND): the role of pain intensity and psychological factors

**DOI:** 10.1007/s00415-026-13817-x

**Published:** 2026-04-15

**Authors:** Lisa Callanan, Fiadhnait O’Keeffe, Tom Burke

**Affiliations:** 1 School of Psychology, University of Galway, Galway, Ireland; 2 School of Applied Psychology, University College Cork, Cork, Ireland; 3 Centre for Neuroimaging, Cognition, and Genomics, University of Galway, Galway, Ireland; 4 University Hospital Galway, Galway, Ireland

**Keywords:** Functional neurological disorder, Pain, Fear-avoidance model, Pain catastrophising, Pain-related disability

## Abstract

**Background:**

Functional neurological disorder (FND) is a complex neurological condition characterised by involuntary motor and sensory symptoms, linked to alterations in brain network functioning rather than abnormalities in brain structure. Pain is common in FND and is associated with reduced quality of life (QoL), distress, and increased disability. The Fear-Avoidance Model (FAM) posits that psychological factors can exacerbate pain and disability. This study aimed to examine whether pain intensity and psychological factors of the FAM predict pain-related disability in individuals with FND. A secondary aim was to assess whether the relationship between pain intensity and pain-related disability was sequentially mediated by pain catastrophising, pain-related avoidance, and depression.

**Methods:**

A total of 248 adults with FND and pain (18–73 years) completed an online cross-sectional survey. Hierarchical regression analysis assessed whether pain intensity and psychological variables predicted pain-related disability. Sequential mediation analysis investigated direct and indirect effects.

**Results:**

Eighty-nine percent of participants reported pain associated with FND, and 93.5% reported persistent pain. Pain intensity, pain catastrophising, and pain-related avoidance explained 58.1% of the variance in pain-related disability. Mediation analysis revealed a significant direct effect of pain intensity on pain-related disability, with partial and sequential indirect effects via psychological variables. The final model explained 61.5% of the variance in pain-related disability.

**Conclusion:**

Participants with FND reported high levels of pain intensity, pain-related disability, and low QoL. These findings highlight the complex interplay between pain and psychological factors in FND, which may be important targets for intervention.

**Supplementary Information:**

The online version contains supplementary material available at 10.1007/s00415-026-13817-x.

## Introduction

Functional neurological disorder (FND) is a common neurological condition characterised by a range of symptoms, including movement difficulties, seizures, sensory disturbances, and/or cognitive complaints diagnosed using positive clinical signs [1–3]. Symptoms of FND are thought to result from dysfunction within and across brain networks rather than structural brain abnormalities [4]. FND places a substantial burden on both individuals and healthcare systems, with frequent acute presentations to emergency departments for both neurological symptoms and pain [5, 6]. Individuals with FND often experience poor outcomes, with symptoms limiting physical activity, employment, social engagement, and straining interpersonal relationships [7]. Physical disability and coexisting psychological difficulties such as low mood and anxiety are common amongst FND cohorts [8, 9]. Quality of life is often reduced to levels comparable to those seen in progressive neurological conditions [10].

FND rarely occurs in isolation, and is often accompanied by pain. According to the International Association for the Study of Pain (IASP, 2020), pain is “an unpleasant sensory and emotional experience associated with actual or potential tissue damage,” but its role in FND remains poorly understood. A recent study reported that up to 84% of individuals with FND experience associated pain [11]. Chronic pain, defined as pain that persists for more than three months is frequently reported in people with FND. A recent systematic review and meta-analysis estimated that 55% of individuals with FND experience chronic pain [12].

Evidence indicates that pain in FND is associated with poorer clinical outcomes including greater severity of weakness [13]. Pain negatively affects health-related quality of life (HRQoL) including mobility, and everyday activities, extending beyond the effects of core FND symptoms such as weakness, gait disturbance, and seizures [14–17]. Higher pain intensity has also been linked to increased anxiety and depression in functional movement disorder (FMD) [15].

Although pain and FND are diagnostically distinct entities, growing evidence highlights shared transdiagnostic mechanisms that contribute to their persistence and chronicity [18–20]. These commonalities include attentional and information-processing biases, escape-avoidance coping strategies, ruminative thinking, difficulties with emotional regulation, somatic preoccupation, heightened autonomic arousal, and threat-related hypervigilance [11, 21–23]. Recognising these shared mechanisms is essential for advancing insight into pain within FND populations.

**Fig. 1:**
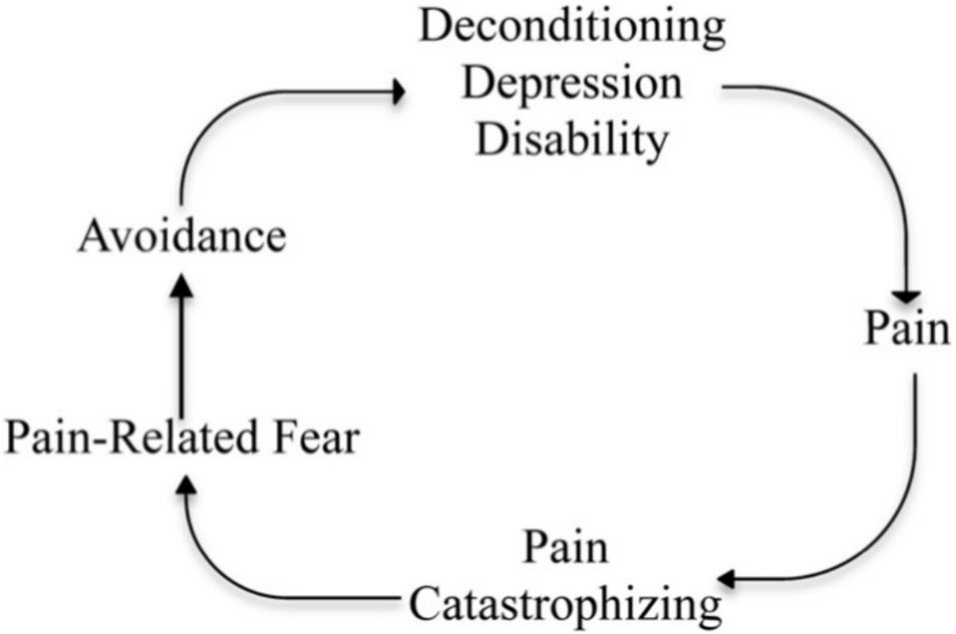
Fear avoidance model of chronic pain (Taken from Kroska et al. [33])

Whilst pain serves an important evolutionary function in protecting the body from acute harm, it can also become maladaptive [24]. A model that offers insight into how pain may shift from a protective signal to a source of disability is the Fear-Avoidance Model (FAM) [25] (Fig. 1). The FAM suggests that when pain is perceived as threatening, particularly in the absence of tissue damage, a cycle of maladaptive cognitive and behavioural responses such as pain catastrophising, fear of pain, and escape-avoidance behaviours may develop. These interrelated processes further amplify pain perception. Whilst catastrophising and avoidance may serve an adaptive role in the short term, their prolonged use can be costly, leading to withdrawal from valued activities, and reinforcing a self-perpetuating cycle of reduced HRQoL, mental health difficulties, physical disuse and chronic disability [26–28]. Current evidence supports the FAM in those with chronic primary pain [29, 30], acute pain [31], and people with persisting symptoms after concussion [32]. The FAM may offer valuable insight into the factors maintaining pain and contributing to its disabling impact in individuals with FND.

Pain catastrophising is a central driver within the FAM [25, 34]. It refers to the tendency to ruminate on aspects of the painful experience, magnify the threat of pain, and feel helpless in response to actual or anticipated painful experiences [35, 36]. More specifically, pain catastrophising involves persistent threat anticipation and dwelling on worst-case scenarios [37]. For example, acute pain from an injury may understandably be perceived as catastrophic. However, persistent catastrophising, such as thoughts like, ‘this pain must mean something serious is wrong’, or ‘If I don’t rest completely, I’ll make this pain much worse and never recover’, can trigger a cascade of pain-related fear and avoidance behaviours [38].

Pain catastrophising is a key psychological factor associated with adverse pain outcomes, including heightened pain sensitivity, increased physical disability, and reduced quality of life in individuals with chronic pain and Parkinson’s disease [39–41]. What is more, greater pain catastrophising is strongly linked to greater emotional distress, including anxiety, fear, and depression in chronic pain [37, 42, 43]. Fearing the worst about pain and perceiving it as catastrophic has also been shown to predict disability outcomes beyond pain intensity in chronic pain [44, 45].

Pain-related fear and behavioural avoidance have also been shown to contribute to greater disability in chronic pain cohorts, particularly in conditions such as complex regional pain syndrome [46]. Some evidence suggests that pain-related fear may predict functional disability beyond the influence of pain intensity [47]. Building on this, a recent systematic review of 27 studies including over 11,000 individuals with chronic shoulder pain found that high levels of emotional distress, depressive symptoms, anxiety, fear-avoidance beliefs, somatisation, and pain catastrophising were significantly associated with increased pain intensity and disability [48]. However, the quality of evidence in this review was rated as very low. A more recent meta-analysis by Rogers and Farris [49] found medium-to-large associations between psychological processes, particularly catastrophic pain cognitions, depression and pain-related functional impairment but found a lesser role for perceived pain intensity.

These inconsistencies in the literature reflect the evolving nature of the FAM, which has undergone revisions in recent years [50]. Whilst pain intensity was not initially considered central to the FAM, a growing body of research has confirmed its critical role on pain-related disability. For instance, research in both general and clinical chronic pain populations demonstrated that perceived pain intensity accounted for 45–67% of the variance in pain-related disability [51–54]. This shift highlights growing acknowledgement that whilst psychological factors such as fear-avoidance and catastrophising are crucial, pain intensity also plays a substantial role in functional impairment [30, 34].

Despite growing interest in the FAM, few studies have examined the sequential interplay of psychological factors within multivariate frameworks. Seekatz et al. [55] aimed to address this gap by investigating the cascading effects of psychological variables on functioning in individuals with chronic low back pain. Their findings revealed small-to-large interrelations between pain intensity, fear-avoidance beliefs, depression, and functioning. Pain intensity had a strong direct effect on functioning, whilst fear-avoidance beliefs contributed indirectly. Depression was also associated with reduced functioning, both directly and through its interaction with fear-avoidance pathways. These findings highlight the multidimensional influence of pain characteristics and psychological processes on pain-related disability in people with chronic pain.

In the context of FND, some components of the FAM have received empirical support, particularly in relation to core functional neurological symptoms. For example, qualitative studies indicate that individuals with FND frequently withdraw from social situations and physical activities to avoid triggering symptoms such as seizures, contributing to increased disability and reduced quality of life [56]. Similarly, Kozlowska et al. [57] found that children with FND often catastrophised and ruminated about their symptoms worsening, and avoided exercise to prevent motor flare-ups, all of which significantly impaired their daily functioning.

Both fear of falling and kinesiophobia (i.e. fear of physical movement and/or (re)injury) [58] have also been linked to lower wellbeing and greater dependent gait patterns (i.e. requiring assistance from another person to walk) in people with functional motor symptoms [59]. Whilst research on fear-avoidance and symptom-related catastrophising in FND is growing, researchers have not yet explicitly examined these psychological processes in relation to pain or their contribution to disability in people with FND.

Pain is a common and burdensome symptom in people with FND, significantly impacting daily functioning and contributing to disability. Although several quantitative studies in the chronic pain literature have identified associations between pain, disability, and psychological factors such as catastrophising, avoidance, and depression, these findings have been inconsistent, with variation in both the strength and direction of these relationships [39, 52].

Whilst aspects of the FAM, particularly catastrophic interpretations and avoidance, have been explored in qualitative studies of people with FND, most have focused on core FND symptoms such as motor difficulties and seizures, rather than pain. As far as we are aware, no published quantitative studies have directly examined these psychological processes in relation to pain in FND. Developing a greater understanding of the role of pain and its psychological correlates on pain-related disability in FND may inform treatment targets. This study seeks to build on existing literature in FND and pain [14, 16, 26, 55] by investigating whether pain intensity, pain catastrophising and pain-related avoidance predict pain-related disability in individuals with FND. The study also aims to examine whether psychological factors mediate the relationship between pain intensity and pain-related disability. Additionally, the study will explore the potential cascade-like process through which pain intensity influences psychological responses that, in turn, affect disability.

This study aimed to examine the following hypotheses:

1. Pain intensity, pain catastrophising and pain-related avoidance will significantly predict pain-related disability in FND.
2. Pain intensity in FND will indirectly affect pain-related disability through a serial chain of mediators, with pain catastrophising, pain-related avoidance, and depression sequentially mediating the relationship.
3. As an exploratory hypothesis, pain and psychological variables will be significantly and negatively associated with HRQoL domains in FND.

## Methods

This cross-sectional online study included participants who self-reported a diagnosis of FND from a healthcare professional. Patient support groups (FND Ireland, FND Friends, FND Australia), organisations (Psychological Society of Ireland’s Division of Neuropsychology, Chronic Pain Ireland, Neurological Alliance of Ireland), and social media platforms (X, Instagram, LinkedIn, Facebook), shared the study poster online. Participants accessed the study survey, hosted on Qualtrics software (Version 24) via a URL link or QR code. Convenience sampling was used due to the self-selecting nature of online participation. The survey took approximately 20–30 min, and data collection commenced on November 1, 2024 and concluded on January 27, 2025. Ethical approval was obtained from the School of Psychology Research Ethics Committee (SREC) at the University of Galway and the Galway Clinical Research Ethics Committee (Approval 2024–10-03). Informed consent was secured from all participants, and this study followed the Declaration of Helsinki. Inclusion criteria included participants 18 years and older, English fluency, and a self-reported FND diagnosis from a medical professional. Exclusion criteria included diagnoses of bipolar, psychotic, or substance misuse disorders; terminal illness; and/or severe cognitive impairment/dementia. These criteria were implemented to reduce potential confounding effects.

### Sample size calculation

An a priori power analysis using G Power (version 3.1) [60] indicated that a sample of 153 participants was required to detect a medium effect size (*f*^*2*^ = 0.15) with an alpha level (*α*) = 0.05 and power = 0.95, based on a regression model with seven predictors (pain intensity, pain catastrophising, pain-related fear, pain-related avoidance, depression and pain-related disability). A medium effect size was selected in accordance with Cohen’s [61] conventions and supported by a previous meta-analysis [49], which reported medium-to-large correlations amongst similar variables.

## Measures

Data collected encompassed demographics (age, gender, country of residence, education level, employment status, and marital status), medical diagnoses, antecedent physical events/injuries, and family history of neurological conditions. The following clinical characteristics were also obtained:

### FND characteristics

With feedback from the FND Ireland research panel, reviewing https://neurosymptoms.org/en/ and previous research [62], five commonly reported FND symptom groups were compiled: movement difficulties, functional seizures, sensory issues, cognitive/memory problems, and swallowing/speech difficulties. Participants indicated their primary FND symptom, with the option to report additional FND symptoms in a subsequent question. Participants reported FND duration, associated FND symptoms, and rated FND symptom severity over the past week on a scale from 0 (none) to 4 (severe).

### Pain characteristics

In line with the IASP, pain that persists or recurs for longer than 3 months was classified as chronic pain [63]. Participants were asked the following questions to assess their pain experiences:

1. Would you consider yourself someone who experiences pain?
2. Has any of this pain that you have experienced lasted longer than 3 months?
3. How long has any pain you have experienced lasted?

### The Brief Pain Inventory–Short Form (BPI-sf)

The Brief Pain Inventory Short Form (BPI-sf) [64] is a widely used self-report measure of pain intensity and pain interference over the past week, using 0–10 numerical rating scales. The pain intensity subscale averages four items (worst, least, average, current pain), whilst the pain interference subscale assesses disability across seven domains: activity, mood, walking, work, relationships, sleep, and enjoyment of life. Both subscales show high test–retest reliability [65], and strong internal consistency [66]. Higher scores reflect greater pain and interference. Pain was classified as mild (1–4), moderate (5–6), or severe (7–10) per Jensen, Smith [67]. In this study, internal consistency was excellent for both subscales: intensity (*α* = 0.93) and interference (*α* = 0.91).

### Pain Catastrophising Scale (PCS)

The Pain Catastrophising Scale (PCS) [35] is a 13-item self-report measure assessing catastrophic thoughts, perceptions and feelings related to pain. Items are rated from 0 (not at all) to 4 (all the time), with total scores ranging from 0 to 52. It includes three subscales: magnification, rumination, and helplessness; however, the total score (sum of all items) was used. Higher scores indicate greater pain catastrophising. The PCS has demonstrated good reliability across pain populations [68, 69]. Internal consistency in the current study was excellent (*α* = 0.95).

### The Pain Anxiety Symptoms Scale (PASS-20)

The Pain Anxiety Symptoms Scale (PASS-20) [70] is a 20-item self-report tool measuring four components of pain-related anxiety: cognitive appraisal, escape/avoidance, fear of pain, and physiological anxiety. Items are rated from 0 (never) to 5 (always), with total scores from 0 to 100 and subscales from 0 to 25. Following prior research aligned with the Fear-Avoidance Model [71], only the fear of pain and escape/avoidance subscales were used in the current study. Internal consistency in the current study was *α* = 0.91 for fear of pain and *α* = 0.83 for escape/avoidance.

### The Patient Health Questionnaire-9 (PHQ-9)

The Patient Health Questionnaire-9 (PHQ-9) [72] is a 9-item self-report measure of depression based on the *Diagnostic and Statistical Manual of Mental Disorders* (5th ed.; DSM-5; American Psychiatric Association, 2013). Items assess symptom frequency over the past two weeks (0 = “not at all” to 3 = “nearly every day”), with total scores ranging from 0 to 27. Cutoffs of 5, 10, 15, and 20 indicate mild to severe depression. A score of ≥ 10 is commonly used to indicate clinically significant depression [73]. The PHQ-9 has shown strong reliability in chronic pain cohorts [74]. In the current study, internal consistency was *α* = 0.86.

### 36-Item Short Form Health Survey (SF-36)

The 36-Item Short Form Health Survey (SF-36) [75] assesses health-related quality of life (HRQoL) across eight domains: physical functioning, role physical, general health, vitality, social functioning, role emotional, mental health, and bodily pain. Bodily pain was excluded to avoid multicollinearity with predictor variables (e.g. pain intensity). Scores were computed using the RAND-36 algorithm [76], transforming responses to a 0 to 100 scale, with higher scores reflecting better HRQoL. Only subscale scores were used to avoid multicollinearity, consistent with recommended practice [77]. Internal consistency for the subscales ranged from *α* 0.72 to 0.92 in the current study.

## Statistical analysis

**Fig. 2:**
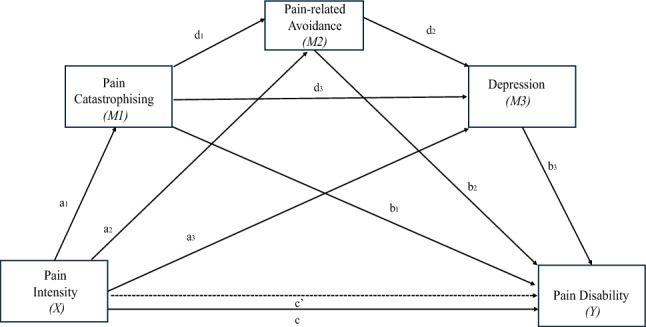
Proposed sequential mediation model. Note. Representation of the proposed sequential multiple mediation model testing the indirect effect of pain intensity on pain-related disability through a cascading pathway involving pain-catastrophising, pain-related avoidance, and depression

IBM SPSS Statistics v29 was used to analyse the data. A *p* value < 0.05 was considered statistically significant. Descriptive statistics are reported as frequencies and percentages for categorical variables and medians, means, ranges, and standard deviations, for continuous variables. Internal consistency of the measures was assessed using Cronbach’s alpha [78], with* α* ≥ 0.7 considered acceptable [79]. Although skewness, kurtosis, and visual inspections of histograms and boxplots indicated approximate normality, the Kolmogorov–Smirnov tests were significant for several continuous variables, therefore, non-parametric tests (i.e. Spearman’s rho and Kruskal–Wallis test) were used to take a more cautious approach. Bonferroni corrections were applied for multiple correlations, with strength interpreted using Cohen’s [80] guidelines: small (*r* = 0.10–0.29), medium (*r* = 0.30–0.49), and large (*r* ≥ 0.50). HRQoL was not a primary variable and was only included in correlation analyses. To evaluate predictors of pain-related disability, a hierarchical regression analysis was conducted. Variables were entered in the way they appear in the FAM model: Block 1) pain intensity, block 2) pain catastrophising, and block 3) pain-related avoidance. Pain-related disability was entered as the outcome variable. Depression was excluded from the regression model to avoid obscuring the unique contributions of catastrophising and avoidance. Additionally, a serial mediation analysis was conducted using PROCESS macro for SPSS (v4.2), Model 6 [81] with bootstrapping (5000 samples). This analysis examined whether the relationship between the independent variable; pain intensity (X) and the dependent variable; pain-related disability (Y) was mediated by a sequence through pain catastrophising (Mediator 1), pain-related avoidance (Mediator 2), and depression (Mediator 3) (Fig. 2). Indirect effects were significant if the 95% confidence interval (CI) did not include zero. Standardised beta coefficients (*β*), standard errors (*SE*), and 95% confidence intervals (CI) are reported.

## Results

### Data screening and normality checks

The data was screened for outliers and normality using visual inspection of histograms, boxplots, kurtosis and skewness values, and case-wise diagnostics. Extreme outliers (*n* = 13), identified from boxplots were removed. The Role Emotional and Role Physical subscales of the SF-36 contained a high number of extreme outliers, which raised concerns about the reliability and interpretability of these variables. These subscales were removed from the study prior to the analysis to maintain data quality. Regression assumptions of linearity, multicollinearity, homoscedasticity, and normality of residuals were assessed. Outliers were evaluated using Mahalanobis distance [82], and three cases with standardised residuals ± 3 were identified. These cases showed Cook’s distances > 1 [83], indicating undue influence on the model, and were therefore removed. Multicollinearity diagnostics indicated acceptable tolerance (0.58–1.0) and variance inflation factor (VIF) values (1.0–1.71) [84]. However, a high intercorrelation (r > 0.70) and elevated VIF between pain catastrophising and pain-related fear suggested shared variance. Pain catastrophising was retained due to its stronger associations with key study variables.

## Descriptive statistics

### Participant demographics

The final study cohort included 248 participants (84.3% female), aged 18–73 years (*M* = 43.62, *SD* = 12.55). No missing data were present for the variables of interest. Table 1 shows the participant demographic and clinical information.

**Table 1:** Demographic characteristics of the study cohort

Characteristics	Total (*N* = 248) *n* (%)
Gender	
Female	209 (84.3)
Male	32 (12.9)
Other	7 (2.8)
Age at survey completion, years, median (IQR, range)	44 (19, 18–73)
Age at FND diagnosis, years, median (IQR)	42 (19)
Country of residence	
Ireland	53 (22.4)
UK	90 (36.3)
Other	104 (41.9)
Prefer not to say	1 (0.4)
Employment status	
At work full time	61 (24.6)
Unemployed	13 (5.2)
Student	9 (3.6)
Retired	12 (4.8)
Engaged in home duties	6 (2.4)
Unable to work due to health reasons	147 (59.3)

### FND characteristics

Clinical characteristics of the 248 participants are detailed in Table 2. Regarding FND phenomenology, over half of the cohort endorsed functional movement difficulties (52.8%) as their primary FND symptom, whilst the remaining participants reported seizures (15.7%), sensory (11.7%), cognitive (8.1%), and swallowing/speech difficulties (2%). Nearly all participants had multiple FND symptoms (*n* = 247), and 128 participants (75.8%) reported having greater than 4 FND symptoms. 83.9% (*n* = 208) rated their FND symptoms as either moderate or severe in the past week. Regarding co-occurring symptoms associated with FND, 93.1% (*n* = 231) reported fatigue and 89.9% (*n* = 223) reported pain.

**Table 2:** FND characteristics of study cohort

FND characteristics	Total (*N* = 248) *n* (%)
Primary FND phenotype	
Movement difficulties	131 (52.8)
Functional seizures	39 (15.7)
Sensory difficulties	29 (11.7)
Cognitive/memory problems	20 (8.1)
Swallowing/speech difficulties	5 (2.0)
Other	24 (9.7)
FND intensity	
None	2 (0.8)
Minimal	8 (3.2)
Mild	30 (12.1)
Moderate	130 (52.4)
Severe	78 (31.5)
FND duration in years	
< 1 year	21 (8.5)
1 to < 5 years	130 (52.4)
5 to < 10 years	43 (17.3)
≥ 10 years	53 (21.4)
Associated FND symptoms, yes, n (%)	248 (100.0)
Anxiety	174 (70.2)
Low mood	166 (66.9)
Fatigue	231 (93.1)
Sleep disturbance	194 (78.2)
Pain	223 (89.9)
Co-occurring neurological disorder, yes, n (%)	68 (27.4)
Family History of neurological condition, yes, n (%)	83 (33.5)
FND physical-precipitating event, yes, n (%)	180 (72.6)

### Pain characteristics

Within the study cohort, 93.5% (*n* = 232) were classified as having chronic pain, whilst 6.5% (*n* = 16) were classified as having acute pain. The most commonly reported pain conditions reported by participants included musculoskeletal pain, migraine, and chronic back pain (Table 3).

**Table 3:** Pain characteristics of study cohort

Pain characteristics	Total (*N* = 248) *n* (%)
Self-reported pain conditions^a^	
Musculoskeletal pain	166 (66.9)
Migraine	153 (61.7)
Chronic back pain	146 (58.9)
Neuropathic pain	142 (57.3)
Irritable Bowel Syndrome	114 (46.0)
Fibromyalgia	73 (29.4)
Chronic pain related to injury, tissue damage, burns, sprains, surgery	61 (24.6)
Sciatica	75 (30.2)
Arthritis	65 (26.2)
Complex Regional Pain Syndrome (CRPS)	15 (6.0)
Cancer-related pain	2 (0.8)
Other	39 (15.7)
Pain identity	
Identifies as someone who experiences pain, yes, n (%)	234 (94.4)
Duration of pain	
Chronic pain ≥ 3 months	232 (93.5)
Acute pain ≤ 3 months	16 (6.5)
Description of nature of pain	
Constant	59 (23.8)
Sudden/intermittent	49 (19.8)
Both	136 (54.8)
BPI-sf pain intensity	
None	8 (3.2)
Mild	89 (35.9)
Moderate	79 (31.9)
Severe	72 (29.0)
Pain medication, yes, *n* (%)	166 (66.9)
Pain physical-precipitating event, yes, *n* (%)	158 (63.7)

^a^cohort presented with a total of 1051 pain conditions, as multiple conditions were endorsed

### Psychological and pain-related variables

Regarding psychological comorbidities, 80.2% (*n* = 199) were classified as having at least moderate depression as per the PHQ-9. Descriptive statistics for predictor, mediator and outcome variables are presented in Table 4.

**Table 4:** Descriptive statistics for pain and psychological measures

Measure	Possible range	Actual range	M (SD)	Mdn (IQR)	Cronbach’s α
Fear avoidance components					
Pain catastrophizing (PCS)	0–52	0–52	23.47 (13.25)	23 (20)	0.95
Pain-related fear (PASS-20)	0–25	0–25	10.50 (7.02)	10 (10)	0.91
Pain-related avoidance (PASS-20)	0–25	0–25	12.84 (6.03)	12.50 (9)	0.83
Pain intensity					
BPI-sf pain intensity	0–10	0–10	5.47 (2.25)	5.75 (3.50)	0.93
Pain-related disability					
BPI-sf pain interference	0–10	0.71–10	6.50 (2.35)	6.71 (3.43)	0.92
Health-related quality of life					
SF-36 domains	0–100				
Physical functioning (PF)	–	0–100	27.50 (23.34)	22.50 (30)	0.91
Vitality (VT)	–	0–70	17.32 (15.93)	15 (20)	0.76
Mental Health (MH)	–	0–96	50.27 (21.20)	52 (32)	0.83
Social Functioning (SF)	–	0–87.50	28.38 (22.76)	25 (37.50)	0.83
General Health (GH)	–	0–85	32.5 (19.04)	30 (25.94)	0.72
Depression					
PHQ9	0–27	1–27	15.14 (6.29)	15 (9.75)	0.86

*N* = 248

*M* mean, *SD* standard deviation, *Mdn* median, *IQR* Interquartile range, *PCS* Pain Catastrophising Scale, *PASS-20* Pain Anxiety Symptom Scale-20 (fear subscale, escape/avoidance subscale), *SF-36* short form, *PF* physical functioning, *VT* vitality (energy and fatigue), *MH* mental health (emotional wellbeing), *SF* social functioning, *GH* general health, *PHQ9* Patient Health Questionnaire-9

### Pain intensity group comparisons

**Fig. 3:**
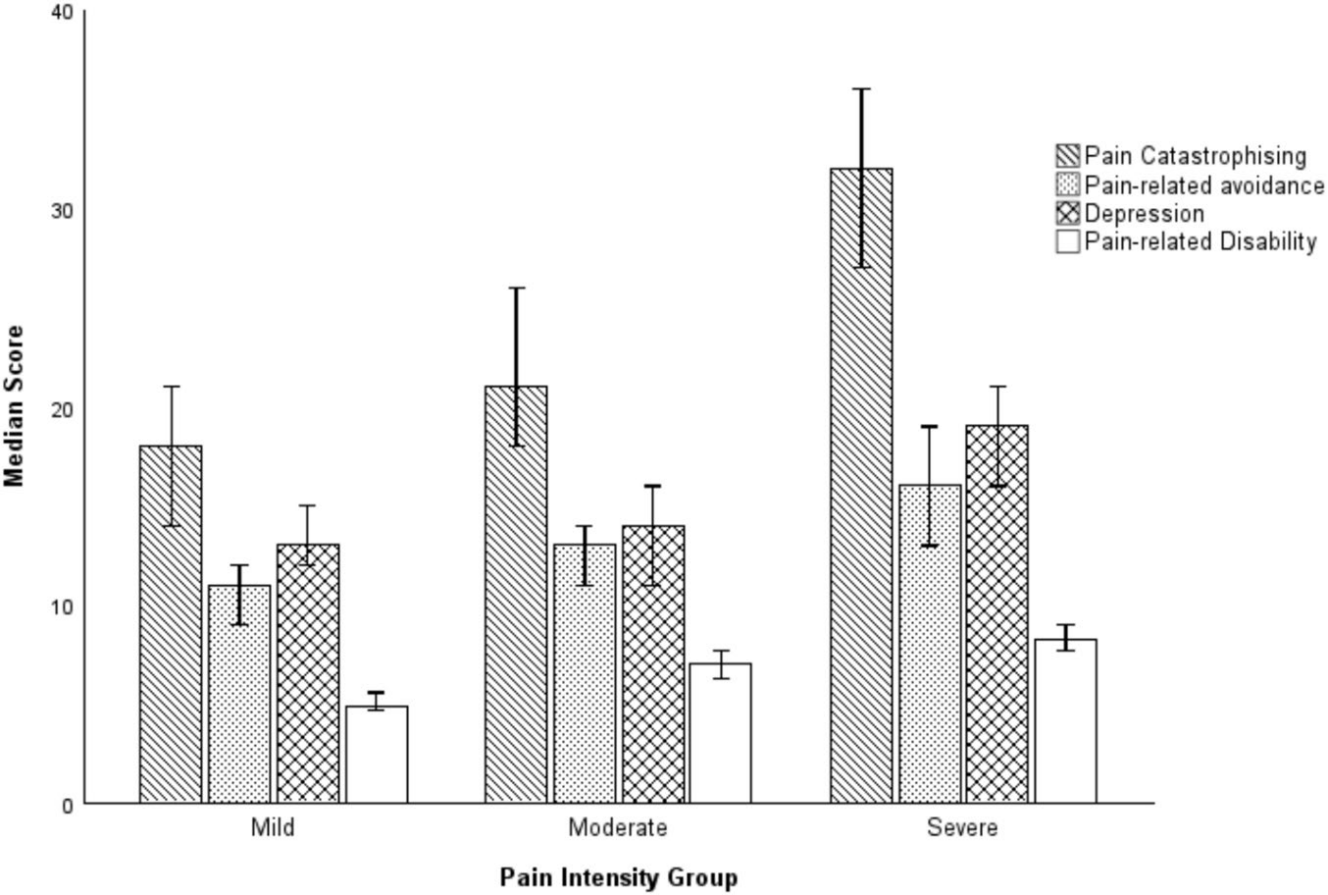
Graphical representation of psychological and pain disability scores across pain intensity groups*. Note.* Error bars represent 95% confidence intervals. Eight participants had pain intensity ratings < 1 and were not included in group comparison analyses

On the BPI-sf pain intensity subscale, 35.9% (*n* = 89) of the total cohort reported mild levels of pain intensity, 31.9% (*n* = 79) reported moderate levels of pain, and 29% (n = 72) reported severe pain. A Kruskal–Wallis test showed statistically significant differences across pain intensity groups for all psychological variables of interest: pain catastrophising; *H*(2) = 32.85, *p* < 0.001, pain avoidance; *H*(2) = 27.84, *p* < 0.001, depression; *H*(2) = 22.01, *p* < 0.001, and pain-related disability; *H*(2) = 85.14, *p* < 0.001. Median scores across groups are shown in Fig. 3.

## Correlational analyses

### Pain-related disability

As seen in Table 5, pain intensity showed significant positive correlations with pain catastrophising *r*_*s*_(246) = 0.38, *p* < 0.001, pain avoidance *r*_*s*_(246) = 0.31, *p* < 0.001, depression *r*_*s*_(246) = 0.34, *p* < 0.001, and pain-related disability *r*_*s*_(246) = 0.70, *p* < 0.001. Pain catastrophising, pain avoidance, and depression showed significant and positive correlations with pain-related disability (ranging from *r*_*s*_ = 0.45 to *r*_*s*_ = 0.53, *p* < 0.001), such that higher levels of psychological variables were associated with higher pain-related disability.

**Table 5:** Spearman Rho correlation matrix amongst predictor and outcome variables

	1	2	3	4	5
1. Pain intensity (BPI-sf pain intensity)	1				
2. Pain catastrophising (PCS)	0.38**	1			
3. Pain avoidance (PASS-20 pain avoidance)	0.31**	0.60**	1		
4. Depression (PHQ-9)	0.34**	0.62**	0.40**	1	
5. Pain disability (BPI-sf pain interference)	0.70**	0.48**	0.45**	0.53**	1

Bonferroni correction *was* applied (adjusted alpha = 0.005)

Correlation is significant at **p < 0.001 (2-tailed)

### Health-related quality of life

Pain intensity showed significant negative correlations with several HRQoL domains, including physical functioning, *r*_*s*_(246) =  − 0.41, *p* < 0.001, social functioning, *r*_*s*_(246) =  − 0.35, *p* < 0.001, and mental health, *r*_*s*_(246) =  − 0.21, *p* < 0.001, such that higher levels of pain intensity were associated with lower levels of HRQoL. Pain catastrophising, pain avoidance, and depression showed significant negative correlations with HRQoL domains, such that higher levels of psychological variables were associated with lower levels of HRQOL. For example, depression was strongly and negatively correlated with HRQoL mental health, *r*_*s*_(246) =  − 0.70, *p* < 0.001, whilst pain catastrophising and pain avoidance also showed moderate negative associations across physical, mental, and social domains of HRQoL (e.g. pain catastrophising and mental health, *r*_*s*_(246) =  − 0.63, *p* < 0.001; pain avoidance and social functioning, *r*_*s*_(246) =  − 0.34, *p* < 0.001). Correlation matrix for associations between pain, psychological variables, and HRQoL domains is shown in Table 1 of Supplementary material.

### Hierarchical multiple regression

In the hierarchical regression model, the three FAM-related variables (pain intensity, pain catastrophising, pain avoidance) were entered as predictors and pain disability as the outcome variable. As shown in Table 6, pain intensity was entered in Block 1, accounting for a significant proportion of variance [*R*^2^ = 0.519, *F*(1, 246) = 265.72, *p* < 0.001]. In Block 2, pain catastrophising contributed significantly to the model [*R*^2^ change = 0.045, *F*(1, 245) = 25.41, *p* < 0.001]. In Block 3, pain avoidance made a significant contribution after controlling for pain intensity and catastrophising [*R*^2^ change = 0.016, *F*(1, 244) = 9.57, *p* = 0.002]. The final model was significant and explained 58.1% of the variance in pain-related disability, *F*(3, 244) = 112.73, *p* < 0.001. Pain intensity had the strongest effect (*β* = 0.620, *p* < 0.001), followed by pain avoidance (*β* = 0.162, *p* < 0.001), and pain catastrophising (*β* = 0.136, *p* < 0.001). Key study variables remained significant after controlling for FND severity.

**Table 6:** Hierarchical multiple regression predicting pain-related disability

	*B*	*SE*	*β*	*ΔR*^*2*^	*R*^2^	95% CI	
						Lower	Upper
*Block 1*				0.519	0.519		
Pain intensity	0.753	0.046	0.721			**0.662**	0.844^***^
*Block 2*				0.045	0.564		
Pain intensity	0.660	0.048	0.632			**0.566**	0.754^***^
Pain catastrophising	0.041	0.008	0.230			**0.025**	0.057^***^
*Block 3*				0.016	0.581		
Pain intensity	0.648	0.047	0.620			**0.555**	0.741^***^
Pain catastrophising	0.024	0.010	0.136			**0.005**	0.043^*^
Pain avoidance	0.063	0.020	0.162			**0.023**	0.104^**^

*B* unstandardised beta coefficient, *β* standardised beta coefficient, *SE* standard error, ΔR^2^ R square change, *R*^2^ cumulative variance accounted for by variables in each block, *CI* confidence interval

Bold = significant at ^***^p < 0.001, ^**^p < 0.01, ^*^p < 0.05

### Multiple sequential mediation analyses

**Fig. 4:**
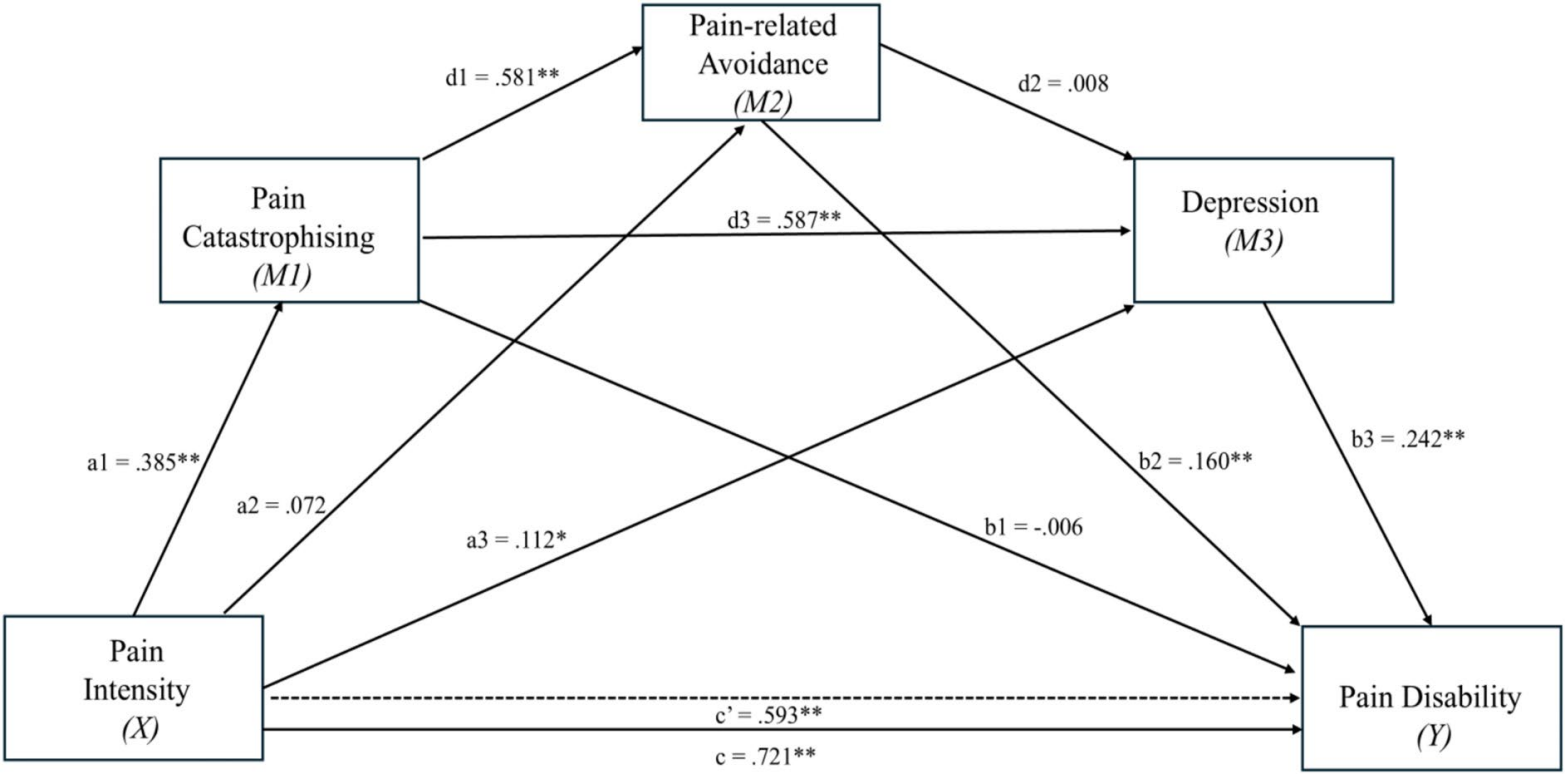
Sequential mediation model of direct and indirect effects on the relationship between pain intensity and pain-related disability*. Note.* Path (**a**) represents the direct effect of pain intensity on each mediator; Path (**b**) represents the effect of each mediator on pain disability; and Path (a*b) represents the indirect effect of pain intensity through each of the individual mediators, whilst Path (a*b*d) represents the indirect effect of pain intensity through two mediators. The magnitude of the indirect effect for each mediator was calculated by the multiplication of the indirect pathways. Path (**c**) represents the total effect of pain intensity on pain-related disability (i.e. equals the sum of the direct effect and the indirect effect(s) in each mediation analysis). Path (**c’**) represents the direct effect of pain intensity on pain disability when the mediators are entered into the model. ^**^significant at *p* < 0.001 level, ^*^significant at *p* < 0.05 level

Sequential mediation analysis revealed several indirect pathways through which pain intensity influenced pain-related disability, with varying magnitudes of effect (Table 7). There was evidence for sequential mediation such that pain catastrophising and pain avoidance (path: a1*d1*b2) mediated the relationship between pain intensity and pain disability, yielding a small effect size *β*indirect = -0.036, *SE* = 0.013, 95% *CI* [0.014, 0.065]. Similarly, the indirect path via pain catastrophising and depression on the relationship between pain intensity and pain-related disability (path: a1*d3*b3) was also significant, with small effect size *β*indirect = 0.055, *SE* = 0.015, 95% CI [0.027, 0.086]. There was an independent indirect effect of depression (path: a2*b3) on the relationship between pain intensity and pain-related disability scores *β*indirect = 0.027, S*E* = 0.016, 95% CI [0.000, 0.063]. The direct effect of pain intensity on pain disability (path c’: *β*direct = 0.593, *p* < 0.001) and the total indirect effect (*β*indirect = 0.127, S*E* = 0.027, 95% CI [0.078, 0.183]) were significant, indicating partial mediation. Indirect paths (via pain catastrophising and avoidance: *β* = 0.036; via pain catastrophising and depression: *β* = 0.055; via depression alone: *β* = 0.027) were significant. The full mediation model was statistically significant, *F*(4, 243) = 97.10, *p* < 0.001, with a large effect size (*R* = 0.784, *R*^*2*^ = 0.615), indicating 61.5% of the variance in pain-related disability was explained by pain intensity, pain catastrophising, pain avoidance and depression. Figure 4 displays the conceptual model illustrating the direct and indirect pathways between pain intensity and pain-related disability. An alternative sequential mediation model was also tested with pain-related disability as the predictor, depression, pain avoidance, and pain catastrophising as mediators, and pain intensity as the outcome. The direct effect of pain-related disability on pain intensity was significant (*β*direct = 0.697, *p* < 0.001). The total indirect effect of pain disability on pain intensity via the psychological mediators was not significant (*β*indirect =  − 0.007, S*E* = 0.034, 95% *CI* [− 0.073, 0.059]), indicating no overall mediation.

**Table 7:** Indirect effects of pain intensity on pain-related disability

Path	Estimate	*SE*	95% CI	
			Lower	Upper
Total indirect effect	0.127	0.027	**0.078**	0.183^*^
a1*b1	− 0.002	0.024	− 0.050	0.048
a2*b2	0.011	0.010	− 0.006	0.035
a3*b3	0.027	0.016	**0.000**	0.063^*^
a1*d1*b2	0.036	0.013	**0.014**	0.065^*^
a1*d3*b3	0.055	0.015	**0.027**	0.086^*^
a2*d2*b3	0.000	0.001	− 0.003	0.003
a1*d1*d2*b3	0.000	0.003	− 0.006	0.008

Bootstrapped standardised estimates and standard errors

Bold^*^ = significant indirect effects

## Discussion

This cross-sectional study investigated the relationship between pain and pain-related disability in FND, and whether this relationship was sequentially mediated by pain catastrophising, pain-related avoidance and depression. Whilst the disabling effects of pain in FND have been reported [14–17], this is, to the authors’ knowledge, the first study to examine the relationship and contribution of pain intensity and the abovementioned psychological factors to disability in individuals with FND.

In the present study, 89.9% of participants reported FND-related pain, slightly higher than previously reported figures in FND cohorts (70–84%) [11, 62, 85], and substantially higher than rates in progressive neurological conditions such as Huntington’s disease (40%) [86]. Nearly 94% of participants reported chronic or persistent pain, exceeding estimates in Parkinson’s disease (e.g. 62%) [41] and in a recent systematic review of FND (e.g. 55%) [12].

Pain was both intense and disabling in the current cohort, with 60% of participants reporting pain intensity in the moderate and severe ranges. These levels are higher than those typically observed in online and self-report samples of individuals with chronic pain or Parkinson’s disease (mean pain intensity ratings of 4) [87, 88]. Rates in the present study are comparable to rates observed in tertiary pain clinics and primary care (68%) [54]. Although studies in multiple sclerosis (MS) have reported higher rates of pain intensity (77.5–85%) [89, 90], these estimates were based on lower BPI-sf cut-offs for classifying moderate pain (e.g. scores of 3–5). Furthermore, 73% of participants in the present study reported at least moderate levels of pain-related disability, with a mean score of 6.5. This exceeds levels reported in primary chronic pain and MS cohorts, where mean scores typically range from 2.2 to 4.8 [51, 71, 91]. The elevated disability levels in this study may reflect the distinct functional challenges experienced by individuals with FND and pain.

In terms of quality of life, HRQoL domains of physical functioning, mental health, energy and fatigue, social functioning, and general health were all notably reduced in the current cohort, consistent with findings in other FND research [7], and markedly lower than primary chronic pain populations [92]. Socioeconomic impacts were also observed, with approximately 60% reporting health-related unemployment, consistent with the literature [93].

Regarding the primary hypothesis, pain intensity and psychological factors together, accounted for 58.1% of the variance in pain-related disability, with pain intensity emerging as the strongest predictor (51.9%), and catastrophising and avoidance behaviours each contributing additional unique variance (i.e. 6.2%). These findings are consistent with chronic pain studies, where pain intensity accounted for 45–67% of pain interference and disability, followed by psychological responses such as ruminative anxiety, catastrophising, psychological distress, and depression [52–54, 71]. The present findings also mirror results in FMD, where pain and depression together explained 58% of perceived disability [14].

The current findings contrast with earlier fear-avoidance models, which emphasised the misinterpretations of pain signals, such as pain-related fear and catastrophising as primary drivers of disability [44, 94]. The present study also challenges conclusions that pain intensity plays only a minor role in the trajectory of disability [95]. Instead, the current findings highlight the combined impact of heightened perceived pain intensity, pain-related cognitions and avoidant behavioural responses in shaping pain-related disability amongst individuals with FND and co-occurring pain.

Regarding the second hypothesis, the mediation model revealed statistically significant pathways to pain-related disability. Psychological factors partially and sequentially mediated the relationship between pain intensity and pain-related disability, contrasting with studies where catastrophising and illness-focussed coping had direct effects [39, 96]. The findings also conflict with those of Ryum and Stiles [45], who also found that the direct effect of pain intensity on disability became non-significant after accounting for catastrophising and fear-avoidance. These discrepancies may reflect variation in outcome measures used. The present study used the Brief Pain Inventory (BPI-sf), which may be sensitive to current and pain-driven functional limitations rather than emotional aspects. Tools like the Pain Disability Index or SF-36 as used in other studies [39, 96] may capture broader, more general or psychologically-influenced aspects of functioning. Additionally, pain intensity ratings were lower in previous samples [39, 96] compared to the current FND cohort, which may further account for these differences.

Beyond measurement differences, another plausible explanation for the strong direct effect of pain intensity on pain-related disability in the present study may lie in its biological function. Pain can be viewed as a hardwired signal of actual or potential bodily threat, with an immediate impact on daily functioning to protect the body from harm [95]. In chronic pain, this signalling system can become dysregulated, making pain function as a false alarm that is difficult to modulate [97]. Over time, this may manifest as an overactive nervous system that becomes hypersensitive to both painful and non-noxious stimuli. Such mechanisms may be relevant in individuals with FND, where recent neuroimaging findings have shown increased activity within sensorimotor networks in people with FND [98], potentially increasing their risk of central sensitisation [99]. Further research into these neurophysiological mechanisms may help guide treatments targeting sensorimotor processing and dysregulation underlying pain perception in FND.

Multiple mechanisms underlie the transition from pain to pain-related disability. In the current study, two key indirect effects emerged. Pain catastrophising and pain-related avoidance partially and sequentially mediated the relationship between pain intensity and disability only when considered together. This may suggest that higher pain intensity increases catastrophising, which in turn promotes avoidance behaviours, reinforcing disability [36]. Disengagement from meaningful activities makes it more difficult to challenge fears and catastrophic interpretations, creating a vicious cycle of avoidance and further functional decline [25]. Over time, restricted physical activity aimed at reducing pain, such as guarding behaviours, excessive rest and/or dependence on others may also lead to kinesiophobia (i.e. fear of movement) and physical consequences like muscle atrophy, stiffness, and deconditioning [100, 101]. These findings suggest that increased pain intensity and threat magnification can together set individuals on a path from pain to disability. This highlights the importance of addressing pain perception and catastrophising as key factors for effective behaviour change.

The findings also support a biopsychosocial model of pain [102], highlighting the intersection of pain and mental health. Pain catastrophising and depression partially and sequentially mediated the relationship between pain intensity and pain-related disability. Specifically, increased pain catastrophising was associated with greater depressive symptoms and worsened functional outcomes. This pattern aligns with previous findings. Wood et al. [103] found that specific components of catastrophising, including magnification and helplessness, partially mediated the relationship between pain intensity and depression in adults with persistent pain. Similarly, individuals with functional seizures showed more perseverative thinking and symptom-related catastrophising than those with epilepsy, correlating with greater depressive symptoms [22].

The indirect effect of catastrophising and depression observed in this study, along with related findings [22, 103, 104], may reflect a process whereby persistent rumination on symptoms interferes with cognitive, emotional, and somatic processing. This mental burden can reduce capacity for adaptive coping and contribute to depressive symptoms [105]. Repeated and unsuccessful attempts to manage or escape from pain may impact one’s sense of self [106], trigger experiences of mental defeat [107], and activate negative self-schemas [108]. Together, these processes may intensify depression, amplify disability, and worsen quality of life.

Furthermore, the mediation model in this study supported an additional path linking pain to disability independently via depression. This aligns with a substantial body of literature identifying depression as a key driver in disabling pain and worsening functioning [55, 109, 110]. Learned helplessness theory [111] offers a useful framework for understanding how persistent pain can lead to feelings of uncontrollability and increase vulnerability to emotional distress [112, 113]. This may be especially salient for individuals with FND, who often report feeling overwhelmed and powerless in response to persistent symptoms [114]. They may also experience a diminished sense of control over their symptoms, grieve the loss of identity or functioning, and feel invalidated and stigmatised within healthcare settings [11, 56, 57, 115]. These experiences can exacerbate helplessness and increase functional impairment. Given the substantial burden that arises when pain and mood difficulties co-occur, there is a pressing need for clinical approaches addressing not only the physical experience of pain but also the emotional responses it evokes.

Moreover, 80.2% of participants in the current study scored in the moderate to severe ranges for depression on the PHQ-9. This is substantially higher than typical rates reported in FND cohorts, which range from 30 to 49% [9, 11, 62, 116]. Depression in the current sample also exceeds the prevalence rates reported by Bulloch et al. [117], which ranged from 18.6% to 33.2% amongst individuals with various neurological conditions, including epilepsy, traumatic brain injury, Alzheimer’s disease, MS, dystonia, and stroke. It is also higher than prevalence estimates from a recent meta-analysis indicating that 39% of adults with chronic pain had clinically significant depressive symptoms [118]. Several factors may account for the higher depression rates observed in this study. The PHQ-9 includes somatic and motor items that may overlap with FND symptoms. Recruitment through peer-support groups may have also introduced self-selection bias, yielding a sample with greater symptom burden. Nonetheless, the elevated prevalence remains notable and may reflect a cohort of individuals with FND experiencing a particularly high symptom severity, one that may be underrepresented in studies using different recruitment or assessment methods.

Regarding HRQoL, medium-to-large associations with pain-related variables were observed. Pain catastrophising and avoidance were strongly associated with lower social functioning and mental health, more so than with physical functioning. These findings are comparable with previous research, indicating that psychological factors are stronger predictors of psychosocial aspects of HRQoL [39, 104]. The biopsychomotor model [119] offers one perspective on how catastrophising functions as a communication behaviour that inadvertently deteriorates social functioning. Catastrophising and other pain behaviours may unconsciously elicit solicitous responses from others, such as encouraging rest or assuming responsibilities on behalf of the person with pain. Whilst well-intentioned, these responses may potentially reinforce fear of pain, reduce participation in occupational, social, and leisure activities, and contribute to social isolation, loneliness, and greater disability [120]. Repeated expressions of pain may also provoke punitive or unsupportive reactions from others, potentially leading to relationship strain and further negatively impacting wellbeing [97]. These significant associations offer preliminary support for the broader psychosocial impact of pain-related processes in individuals with FND and pain.

This study is one of the few to examine psychological factors underlying pain and pain-related disability in people with FND. By exploring these processes, the findings challenge lingering mind–body dualism and support a more integrated, biopsychosocial understanding of FND and pain. The study has several key strengths. The online recruitment strategy enabled international participation and produced a large, well-powered sample. This supported complex statistical modelling and reduced the risk of Type II errors. The inclusion of a clinically-heterogeneous sample, encompassing individuals with varying pain trajectories and comorbid neurological conditions enhances ecological validity and increases the generalisability of findings to real-world clinical settings. Finally, incorporating PPI patient involvement ensured the research remains relevant and meaningful to those with lived experience of FND and pain.

## Limitations and future directions

This study has several limitations. First, the cross-sectional design means the proposed mediation pathway should be interpreted in a statistical rather than causal sense, providing only a snapshot of the relationships amongst variables. Although the mediation model was specified in accordance with the sequential processes outlined in the FAM, reverse or reciprocal pathways are also plausible. For instance, pain-related disability may increase depression, avoidance, and catastrophising, which could in turn amplify pain intensity [40, 121]. To explore this, we tested an alternative model in which pain-related disability preceded the psychological variables and subsequently pain intensity. This model did not yield a significant indirect effect, providing no support for mediation in that direction. Whilst these analyses cannot establish causality, they offer preliminary evidence for the hypothesised ordering of FAM components. Longitudinal research is needed to clarify temporal precedence and the dynamic interplay amongst these relationships.

Additionally, the analysis did not differentiate between pain associated with FND and other pain conditions, and future studies could explore how types of pain impact psychological and functional outcomes in FND. The reliance on self-report measures introduces potential bias, including shared method variance, recall bias, and social desirability effects [122]. Data were collected via online recruitment without embedded quality control measures, increasing the risk of spurious responses that may compromise data reliability and validity. Future studies should consider incorporating attention checks and other quality assurance procedures to enhance data integrity. Objective measures of avoidance, such as diary entries or activity monitoring, could also be included to complement self-report data.

Participants self-identified as having been diagnosed with FND and volunteered for the study. Whilst this facilitated a large sample size for model testing, it introduced potential self-selection bias, with participants possibly being more motivated to participate or experiencing more severe symptoms. Furthermore, the sample was predominantly female, which, although representative of typical gender proportions in FND, may limit the generalisability of findings to other gender identities. Excluding participants with bipolar disorder, psychosis, and substance misuse disorders further restricts generalisability of findings to those with more complex mental health presentations. Sociodemographic and clinical variables (e.g. employment status, age, medications, physical comorbidities, fatigue) were not controlled for, despite their potential influence on pain perception and perceived disability [52]. Although this was intended to avoid overcontrol bias [123], confounding cannot be ruled out.

In this study, pain intensity, pain catastrophising, pain-related avoidance, depression, and pain-related disability were examined as components of the FAM. Fear of pain was not included due to its very high correlation with pain catastrophising, which raised concerns about multicollinearity and construct overlap. Identifying a tool that captures ‘fear-avoidance’ simultaneously also proved difficult in the present research process. Therefore, only a partial FAM was tested. To fully evaluate the FAM, future studies would need to incorporate measures of pain-related fear and/or fear-avoidance. Future research could also examine whether psychological processes, like those in this study, vary across different FND phenotypes. Pain-related disability is multifactorial and is shaped by a range of factors beyond those included in this study. Future research could consider additional biological (e.g. muscle atrophy), neurophysiological (e.g. nociception, functional brain connectivity), and psychosocial (e.g. psychological flexibility, social support) factors. Investigating these factors may reveal their buffering effects on the impact of pain-related disability in FND. This may also reveal additional personalised treatment targets beyond those identified in the current study.

## Clinical implications

The findings have several important clinical implications. Pain is frequently overlooked in FND care, where attention often centres on core neurological symptoms [62, 99, 124]. Yet, this study shows that pain is both very common and debilitating in people with FND, significantly impairing quality of life and daily functioning. Clinicians should routinely assess non-motor symptoms, such as pain and fatigue, and integrate them into person-centred treatment plans to support recovery more holistically. Routine screening for mood disorders is also recommended in FND due to their high co-occurrence (80.2%), and impact on functioning and overall well-being. Pain catastrophising should also be considered a relevant therapeutic psychological treatment target in FND.

Psychological therapies such as Cognitive Behavioural Therapy (CBT), Acceptance and Commitment Therapy (ACT), and mindfulness-based approaches have been effective in reducing pain catastrophising and depression, whilst improving functioning, psychological flexibility, and pain acceptance in adults with chronic pain [125]. Similarly, CBT-based approaches have been shown to reduce FND symptom severity, psychological distress, and fear-avoidance patterns in FND, whilst also improving HRQoL [126, 127]. These improvements were achieved through challenging catastrophic symptom expectations and enhancing the individual’s sense of agency over their FND symptoms. However, these approaches have not yet been specifically evaluated for their effects on pain in FND.

Given the high rates of pain intensity and their direct impact on pain-related disability in this FND cohort, using pain neuroscience-based education to reconceptualise pain, in conjunction with physiotherapy and/or CBT may also favourably influence the cognitions and beliefs about pain, and reduce the ‘extra sensitive’ nature of the nervous system in people with FND [128, 129]. Additionally, future clinical trials in FND should include pain outcome measures and people with co-occurring pain, regardless of intensity, to improve treatment equity and planning. This is especially important given that previous treatment trials in FND have often excluded participants reporting severe pain [130, 131].

An important clinical consideration involves how psychological terms are communicated. The term pain catastrophising, although well-established, has drawn criticism for implying blame or exaggeration [37, 132]. Clinicians should use language that invites patients to share their pain experiences in a non-stigmatising way [132]. Framing discussions in a validating, empathic, collaborative way may enhance the therapeutic relationship, promote engagement and optimise recovery [133, 134].

Although pain is not a core diagnostic criterion for FND [19], its high prevalence and clinical impact raise important questions about how FND and associated pain should be conceptualised, whether as distinct co-occurring conditions or as interconnected manifestations of a broader functional syndrome. Regardless of classification, pain should be routinely considered in diagnostic formulation, assessment, and treatment planning. Integrating pain into care pathways may support more targeted referrals and promote a transdisciplinary approach involving neurology, neuropsychology, physiotherapy, occupational therapy, and pain management specialists, ultimately improving outcomes for people with FND and pain.

In conclusion, pain is a common and often debilitating symptom for people living with FND. Very high levels of pain-related disability and impaired HRQoL were identified. Pain-related disability was predicted by higher levels of pain intensity, pain catastrophising, pain-related avoidance, and depression. Sequential mediation analyses provided significant direct and indirect effects, offering support for current models on pain, specifically components of the FAM in an FND cohort. These findings highlight the need to consider both pain and its psychological correlates in FND, to guide holistic, multimodal interventions. Future research could explore potential protective factors and additional outcomes to further enhance understanding and to develop clinical approaches when supporting individuals with FND and pain.

## Supplementary Information

Below is the link to the electronic supplementary material.

Supplementary file1 (DOCX 24 KB)

## Data Availability

The data that support the findings of this study are available from the corresponding author, upon reasonable request.
