# Supplementary material for: Pain-related disability in functional neurological disorder (FND): the role of pain intensity and psychological factors
Source: J Neurol. 2026 Apr 15;273(5):273. doi: 10.1007/s00415-026-13817-x (PMC13083420; doi:10.1007/s00415-026-13817-x)
Supplement: Supplementary file 1 — Supplementary file1 (DOCX 24 KB) [file 415_2026_13817_MOESM1_ESM.docx]

**Table S1** Spearman Rho Correlations Matrix among pain-related variables and Health-Related Quality of Life

|  | **1** | **2** | **3** | **4** | **5** | **6** | **7** | **8** | **9** |
| --- | --- | --- | --- | --- | --- | --- | --- | --- | --- |
| **1. Pain Intensity (BPI sf)** | **1** |  |  |  |  |  |  |  |  |
| **2. Pain Catastrophising (PCS)** | .38** | **1** |  |  |  |  |  |  |  |
| **3. Pain Avoidance (PASS-20)** | .31** | .60** | **1** |  |  |  |  |  |  |
| **4. Depression (PHQ-9)** | .34** | .62** | .40** | **1** |  |  |  |  |  |
| **5. Physical Health (PH)** | -.41** | -.20** | -.26** | -.36** | **1** |  |  |  |  |
| **6. Vitality (VT)** | -.12 | -.30** | -.29** | -.48** | .36** | **1** |  |  |  |
| **7. Mental Health (MH)** | -.21** | -.63** | -.39** | -.70** | .14* | .40** | **1** |  |  |
| **8. Social Functioning (SF)** | -.35** | -.45** | -.34** | -.58** | .44** | .39** | .40** | **1** |  |
| **9. General Health (GH)** | -.16 | -.33** | -.21** | -.27** | .22** | .35** | .33** | .21** | **1** |

*Note.* Bonferroni correction correlations (adjusted alpha = 0.0014). Correlation is significant at *******p* <0.001 (2-tailed).

PCS; Pain Catastrophising Scale, PASS-20; Pain Anxiety Symptom Scale-20 (escape/avoidance subscale), SF-36; Short Form: PF; Physical Functioning, VT; Vitality (energy & fatigue), MH; Mental Health (emotional wellbeing), SF; Social Functioning, GH; General Health, PHQ9: Patient Health Questionnaire-
